# Practical Medicine

**Published:** 1877-08

**Authors:** 


### Practical Medicine.

Aneurisms on the Terminal Branches of the Pulmonary artery. B. Bramwell. (*Edinburg Med. Journal*, May.) B. reports two cases of fatal hæmoptysis, in which the condition of the lungs, at post mortem examination, seemed to confirm the statements of Rasmussen and others, that in many instances, the source of the hæmorrhage is a ruptured aneurism of a terminal branch of the pulmonary artery.

The first patient, a colored man, æt. 21, suffered from extensive pulmonary disease, more marked on the right than on the left side.

While in hospital, he had three terrible attacks of hæmorrhage, expectorating on each occasion between thirty and forty ounces. The man died some thirty hours after the last bleeding. At the autopsy was found extensive tubercular disease of both lungs. The right apex was hollowed out into a cavity the size of an orange. At the inferior part of this cavity, the walls of which were dense and cicatricial, there was a small aneurism the size of a pea. It was unruptured and was situated on a minute branch of the pulmonary artery. A second smaller cavity in the middle of the lung was filled with coagulated blood, partly recent, partly old and discolored.

A second aneurism, the size of a cherry, was found in the cavity. The aneurism had ruptured, by a slit-like opening, about three lines in length.

This aneurism was evidently the source of the hæmorrhage. Firmly adherent to the outer surface of the sac, and partly blocking the ruptured orifice, was a portion of the decolorized clots. Still lower down there was a third aneurism larger than the others and more irregular in shape.

This aneurism almost completely filled the cavity in which it was situated. The wall was at one point very thin, but it was unruptured. It was situated on a fair-sized branch of the pulmonary artery. The cavity of the aneurism was divided by a well-marked septum into two parts. From the lower end of the sac two efferent vessels sprang.

The second patient, a man æt. 28, suffered from tubercular phthisis. On December 15th, he was attacked with violent hæmoptysis, the blood spirting from the mouth and nose. He was dead in a few minutes. At the post mortem, both lungs were found infiltrated with tubercle. In the left apex there was a cavity the size of a small apple, and in that cavity was situated a round aneurism the size of a cherry.

The aneurism had ruptured by an opening sufficiently large to admit the passage of a good sized probe.

The cavity in which it was contained, the bronchial tubes of both lungs, and the trachea were filled with blood, partly fluid, partly coagulated.

All four aneurisms contained a small quantity of fluid blood. In none was a clot found.

Three Cases of Dysentery Treated Successfully by Large Doses of Ipecacuanha — Given by the Non-Emetic Plan. Forester. (*The Boston Med. and Surg. Jour.* Feb. 1877.) The following cases of dysentery treated by ipecac are of peculiar interest, says the author, because of the prevalent notion that large doses of the drug cannot be given beneficially to dysenteric patients without being followed by emesis. Case One was treated by twenty-five grains of pulverized ipecac every six hours, suspended in syrup of orange peel, and patient instructed to remain in the horizontal position, and to abstain from food and liquids during the treatment.

The ipecac, if rejected, to be repeated every twenty minutes until retained. The other cases reported were treated as the first, and the result obtained in each was speedy convalesence, followed by recovery. W. F. L.

TREATMENT OF FISSURES OF THE NIPPLES DURING LACTATION. Buttler. (*The Ohio Med. Record.* May, 1877.) When fissures of the nipples are not due to some constitutional cause, tinct. of benzoin freely applied to the parts will, in about five to ten days, effect a cure. Only the first application is painful. Tinct. of benzoin forms a covering on the surface of the nipple, and so protects it from the child. Lactation is never interrupted by the above process of treatment.

